# Pulse pressure variation and volume responsiveness during acutely increased pulmonary artery pressure: an experimental study

**DOI:** 10.1186/cc9080

**Published:** 2010-06-24

**Authors:** Fritz Daudel, David Tüller, Stefanie Krähenbühl, Stephan M Jakob, Jukka Takala

**Affiliations:** 1 Department of Intensive Care Medicine, University Hospital (Inselspital) and University of Bern, Freiburgstrasse, CH-3010 Bern, Switzerland

## Abstract

**Introduction:**

We found that pulse pressure variation (PPV) did not predict volume responsiveness in patients with increased pulmonary artery pressure. This study tests the hypothesis that PPV does not predict fluid responsiveness during an endotoxin-induced acute increase in pulmonary artery pressure and right ventricular loading.

**Methods:**

Pigs were subjected to endotoxemia (0.4 μg/kg/hour lipopolysaccharide), followed by volume expansion, subsequent hemorrhage (20% of estimated blood volume), retransfusion, and additional stepwise volume loading until cardiac output did not increase further (n = 5). A separate control group (n = 7) was subjected to bleeding, retransfusion, and volume expansion without endotoxemia. Systemic hemodynamics were measured at baseline and after each intervention, and PPV was calculated offline. Prediction of fluid-challenge-induced stroke volume increase by PPV was analyzed using receiver operating characteristic (ROC) curves.

**Results:**

Sixty-eight volume challenges were performed in endotoxemic animals (22 before and 46 after hemorrhage), and 51 volume challenges in the controls. Endotoxin infusion resulted in an acute increase in pulmonary artery and central venous pressure and a decrease in stroke volume (all *P *< 0.05). In endotoxemia, 68% of volume challenges before hemorrhage increased the stroke volume by > 10%, but PPV did not predict fluid responsiveness (area under the ROC curve = 0.604, *P *= 0.461). After hemorrhage in endotoxemia, stroke volume increased in 48% and the predictive value of PPV improved (area under the ROC curve for PPV = 0.699, *P *= 0.021). In controls after hemorrhage, stroke volume increased in 67% of volume challenges and PPV was a predictor of fluid responsiveness (area under the ROC curve = 0.790, *P *= 0.001).

**Conclusions:**

Fluid responsiveness cannot be predicted with PPV during acute pulmonary hypertension in porcine endotoxemia. Even following severe hemorrhage during endotoxemia, the predictive value of PPV is marginal.

## Introduction

Fluid challenges are frequently used to treat hemodynamically unstable patients, in order to enhance cardiac function by increasing preload. Once the flat part of the cardiac function curve has been reached, the patients are no longer volume responsive [1]. In such cases, further fluid administration can be detrimental due to unnecessary loading of the heart, increased tissue edema, and consequent risk of impaired tissue perfusion.

Cyclic variations of intrathoracic pressure during mechanical ventilation induce acute alterations in cardiac preload and afterload, and are reflected in arterial pressure. Several studies have proposed that pulse pressure variation (PPV) can be used to predict volume responsiveness in mechanically ventilated patients [2-4]. In hypovolemia, the heart operates on the steep part of the cardiac function curve. Hence, the preload reduction induced by positive inspiratory pressure should enhance stroke volume variation and PPV. This hypothesis has been demonstrated in experimental studies [5] and in patients [2,3,6-8], and has been widely adopted in clinical practice to guide fluid therapy.

False-positive predictions of fluid responsiveness with PPV are not uncommon in clinical practice. Acute right ventricular dysfunction can also increase PPV if the increase in afterload due to positive intrathoracic pressure is more relevant than the concomitant reduction in venous return [9]. A failing right ventricle may also impair left ventricular filling during inspiration. A clinically relevant number of false-positive PPVs have been reported recently in critically ill patients with right ventricular dysfunction [10].

Acute right ventricular failure is common in intensive care unit patients, and may occur in about one-third of patients with septic shock [11,12]. In an accompanying paper, we have shown that PPV does not predict fluid responsiveness in critically ill patients with increased pulmonary arterial pressure [13]. The aim of the present study was to validate these findings in pigs in which pulmonary artery pressure was acutely increased by endotoxin infusion.

## Materials and methods

The study was performed in accordance with the National Institutes of Health guidelines for the care and use of experimental animals, and with the approval of the Animal Care Committee of the Canton of Bern, Switzerland.

### Anesthesia and monitoring

Thirteen pigs (body weight 37 to 48 kg, five females) were deprived of food but not of water for 24 hours before the experiments. They were premedicated with atropine 0.05 mg/kg body weight and azaperon (Stresnil^®^; Janssen Pharmaceutica, Beerse, Belgium) 4 mg/kg intramuscularly, followed by cannulation of an ear vein and intravenous administration of 8 to 10 mg/kg pentobarbital (Vetanarchol^®^; Veterinaria AG, Zürich, Switzerland) for endotracheal intubation 5 minutes later. Anesthesia was maintained with pentobarbital 6 to 12 mg/kg/hour and fentanyl 30 μg/kg/hour until the end of the operation.

After the end of the preparation phase, fentanyl was reduced to 5 μg/kg/hour. Neuromuscular blockade was maintained by continuous infusion of pancuronium (Pavulon^®^; Organon, Pfäffikon, Switzerland) to suppress spontaneous breathing and to avoid shivering. The animals were ventilated with a volume-controlled ventilator (Servo 900C; Siemens, Erlangen, Germany) with 5 cmH_2_O positive end-expiratory pressure. FIO_2 _was adjusted to keep PaO_2 _levels between 100 mmHg (13.3 kPa) and 150 mmHg (20 kPa), and remained constant throughout the experiment. The tidal volume was kept at 10 ml/kg and the minute ventilation was adjusted to maintain PaCO_2 _levels between 34 and 41 mmHg (4.5 to 5.5 kPa); after initial adjustment of minute ventilation, the tidal volume was kept constant during the experiment. During animal preparation, 150 ml hydroxyethyl starch (Voluven 6%; Fresenius Kabi AG, Stans, Switzerland) was given in all pigs. Blood losses were substituted additionally with hydroxyethyl starch.

### Animal preparation

After induction of anesthesia, the carotid artery and femoral and jugular veins were exposed surgically. A pulmonary artery catheter (CO/SvO_2 _Catheter; Edwards Lifesciences, Irvine, CA, USA) was inserted via the jugular vein under pressure monitoring. A carotid artery catheter and a femoral venous large bore intravascular sheet for fluid removal and administration were inserted.

### Hemodynamic monitoring and data recording

Intravascular pressures were recorded with quartz pressure transducers, displayed continuously on a multimodular monitor together with the airway pressure (S/5 Critical Care Monitor; Datex-Ohmeda, Helsinki, Finland), and recorded on a computer at a sampling rate of 300 Hz (S-Collect software; Datex-Ohmeda). All pressure transducers were calibrated simultaneously and were zeroed to the level of the heart. Cardiac output was measured using the thermodilution technique (mean value of three bolus measurements using cold saline boluses). The heart rate was measured from the continuously monitored electrocardiogram, and the stroke volume was calculated by dividing cardiac output by the heart rate. After each step of bleeding and each volume challenge, hemodynamic variables were recorded for data analysis.

### Experimental protocol

After preparation and catheter insertion, 30 minutes were allowed for hemodynamic stabilization. An infusion of Ringer's lactate (Sintetica-Bioren SA, Couvet, Switzerland) was set at 2 ml/kg throughout the experiment. After baseline measurements, endotoxin (*Escherichia coli *lipopolysaccharide B0111:B4; Difco Laboratories, Detroit, MI, USA) was infused in the right atrium of five animals at an initial rate of 0.4 μg/kg/hour until the mean pulmonary artery pressure reached two-thirds of the mean systemic pressure. The infusion was then stopped and subsequently adjusted to maintain moderate pulmonary hypertension (mean pulmonary artery pressure, 30 to 35 mmHg). Hydroxyethyl starch (Voluven 6%; Fresenius Kabi AG) was rapidly injected using a 50 ml syringe in boluses of 10% of the estimated blood volume (75 ml/kg [14]) as long as the cardiac output increased > 10%. Volume loading was stopped when two consecutive volume challenges showed no increase in cardiac output > 10%.

Subsequently, the animals were bled by increments of 10% of their estimated blood volume up to a blood loss of 20%. Bleeding was aborted when the systolic blood pressure was below 45 mmHg or the cardiac output was below 1.5 l/minute. The shed blood was then retransfused and additional volume challenges were administered in the form of hydroxyethyl starch in portions of 10% of the estimated blood volume, until cardiac output did not increase further. At the end of the experiment, the animals were sacrificed with an injection of 20 mmol potassium chloride.

A protocol of bleeding up to a blood loss of 20%, retransfusion and further volume expansion was also performed in a separate control group of eight animals.

### Analysis of arterial pressure waveforms

The pressures were analyzed offline. Systolic and diastolic arterial pressures were measured on a beat-to-beat basis, and the pulse pressure was calculated as the difference between systolic and diastolic pressures. Maximal and minimal systolic pressures (*P*_s max _and *P*_s min_) and pulse pressures (*P*_p max _and *P*_p min_) were determined over a single respiratory cycle. PPV was calculated as [3]:

End-tidal carbon dioxide and airway pressure signals were used to define the respiratory cycle.

### Evaluation of volume response

Changes in stroke volume were used to define response to volume challenge. An increase in stroke volume ≥10% following volume administration was considered a positive response. The volume challenge should increase the stroke volume as a result of acutely increased preload in a heart operating on the steep portion of the cardiac function curve. The results were therefore analyzed in two ways: including all volume challenges, and including only those resulting in an increase in central venous pressure (CVP) > 1 mmHg.

### Statistical analysis

The SPSS for Windows 12.0.1 software package (SPSS Inc., Chicago, IL, USA) was used for statistical analysis. Distribution characteristics were assessed using the Kolmogorov-Smirnov test. Data are expressed as mean ± standard deviation if not stated otherwise. Comparison of several means was performed using repeated-measures analysis of variance and Scheffe's test for *post hoc *analysis. The effects of fluid administration on hemodynamic parameters were assessed using the paired *t *test or the Wilcoxon rank sum test. Proportions were compared using Fisher's exact test. Receiver operating characteristic (ROC) curves were constructed to evaluate the predictive value of PPV. The best predictive threshold was defined as the highest sum of sensitivity and specificity. In addition, the predictive value of a PPV threshold of 13% was also evaluated. Data are presented as percentages (proportional data) and as mean ± standard deviation (hemodynamic variables). *P *< 0.05 was considered statistically significant.

## Results

One animal from the group with bleeding and without endotoxemia died during the first step of bleeding due to ventricular fibrillation, and was therefore excluded. All pigs in the endotoxin group tolerated the lipopolysaccharide dose of 0.4 μg/kg/hour. Endotoxin infusion resulted in tachycardia, increased pulmonary artery and central venous pressures, and decreased stroke volume (all *P *< 0.05), but no change in PPV (Table 1). During the volume expansion a total of 1,250 ± 160 ml fluid was infused, followed by 1,540 ± 150 ml of bleeding. Subsequent retransfusion and further volume loading added up to 2,660 ± 560 ml. In the control group, a total of 600 ± 80 ml fluid was bled, followed by retransfusion and further volume loading for a total of 2,260 ± 280 ml. The hemodynamics are summarized in Table 1.

**Table 1:** Overview of systemic hemodynamic values and blood pressure variation during the whole study protocol

		Baseline	Endotoxin	Baseline for bleeding (after volume expansion)	Bleeding	Retransfusion (after last volume challenge)
HR (beats/min)	Endotoxin	93 ± 14	111 ± 5*	107 ± 9	133 ± 17	112 ± 12
	Control	124 ± 18			119 ± 18	127 ± 9
BPm (mmHg)	Endotoxin	78 ± 10	69 ± 12	97 ± 17	37 ± 12	111 ± 31^†^
	Control	68 ± 10			38 ± 7	111 ± 19^‡^
PAOP (mmHg)	Endotoxin	8 ± 1	9 ± 2	16 ± 5	7 ± 1	18 ± 5^†§^
	Control	5 ± 1			4 ± 2	14 ± 2^‡^
CVP (mmHg)	Endotoxin	6 ± 1	8 ± 2*	17 ± 2	5 ± 2	19 ± 4^†§^
	Control	4 ± 1			3 ± 2	12 ± 1^‡^
PAPm (mmHg)	Endotoxin	18 ± 1	45 ± 6*	46 ± 4	28 ± 3	47 ± 7^†^
	Control	14 ± 3			11 ± 2	28 ± 4
SvO_2 _(%)	Endotoxin	63 ± 7	55 ± 10	64 ± 9	33 ± 18	64 ± 9^†^
	Control	49 ± 5			31 ± 5	69 ± 3^‡^
CO (l/min)	Endotoxin	4.4 ± 0.8	2.6 ± 0.9**	5.7 ± 1.0	2.0 ± 0.5	5.8 ± 0.7^†§^
	Control	3.4 ± 0.7			2.1 ± 0.8	9.4 ± 1.9^‡^
SV (ml)	Endotoxin	48 ± 11	24 ± 8*	53 ± 9	15 ± 4	51 ± 2^†§^
	Control	29 ± 11			18 ± 9	74 ± 16^‡^
PPV (%)	Endotoxin	13 ± 4	10 ± 4	8 ± 4	27 ± 9	9 ± 3^†^
	Control	11 ± 5			26 ± 7	7 ± 5^‡^
SPV, Δup (mmHg)	Endotoxin	4 ± 0	2 ± 1	2 ± 0	2 ± 1	2 ± 0
	Control	2 ± 1			3 ± 2	2 ± 2
SPV, Δdown (mmHg)	Endotoxin	3 ± 1	2 ± 1	1 ± 1	5 ± 3	2 ± 1
	Control	5 ± 3			4 ± 7	6 ± 2

Data represent the mean ± standard deviation at the end of each phase. HR, heart rate; BPm, mean arterial blood pressure; PAOP, pulmonary artery occlusion pressure; CVP, central venous pressure; PAPm, mean pulmonary artery pressure; SvO_2_, mixed venous oxygen saturation; CO, cardiac output; SV, stroke volume; PPV, pulse pressure variation; SPV, systolic pressure variation. *P < 0.05, **P = 0.06 compared with baseline. ^†^*P *< 0.05 compared with endotoxin plus bleeding. ^‡^*P *< 0.05 compared with bleeding. ^§^*P *< 0.05 compared with endotoxin (paired *t *test). ^¶^*P *< 0.05 compared with baseline.

Sixty-eight fluid challenges were performed during endotoxemia (22 before bleeding and 46 during retransfusion and volume expansion; Table 2), and 37 of these (54%) increased stroke volume. If only fluid challenges that increased the CVP by > 1 mmHg are considered (n = 60), then 34 (57%) challenges increased the stroke volume.

**Table 2:** Prediction of increase in stroke volume based on receiver operating characteristic curves

	Fluid challenges (*n*)	Responders, *n *(%)	Nonresponders, *n *(%)	AUC (95% CI)	*P *value	Best PPV threshold (%)^a^
All fluid challenges in endotoxemia	68	37 (54)	31 (46)	0.642 (0.505 to 0.778)	0.045	12
Volume expansion before bleeding	22	15 (68)	7 (32)	0.610 (0.365 to 0.854)	0.418	10
After bleeding during retransfusion and volume expansion	46	22 (48)	24 (52)	0.699 (0.543 to 0.854)	0.021	9
All fluid challenges in endotoxemia with CVP increase	60	34 (57)	26 (43)	0.633 (0.485 to 0.780)	0.080	9
Volume expansion before bleeding	20	14 (70)	6 (30)	0.595 (0.335 to 0.855)	0.509	9
After bleeding during retransfusion and volume expansion	40	20 (50)	20 (50)	0.698 (0.528 to 0.867)	0.033	9
All fluid challenges in controls after bleeding	51	34 (67)	17 (33)	0.79 (0.664 to 0.915)	0.001	9^b^
All fluid challenges in controls after bleeding with CVP increase	39	28 (72)	11 (28)	0.724 (0.556 to 0.892)	0.031	11

AUC, area under the receiver operating characteristic curve; CVP, central venous pressure; CI, confidence interval; PPV, pulse pressure variation. ^a^Best PPV thresholds calculated despite *P *values that are not significant. ^b^Threshold for controls is the mean value between two with identical sum of sensitivity and specificity (8% and 10%).

**Figure 1:**
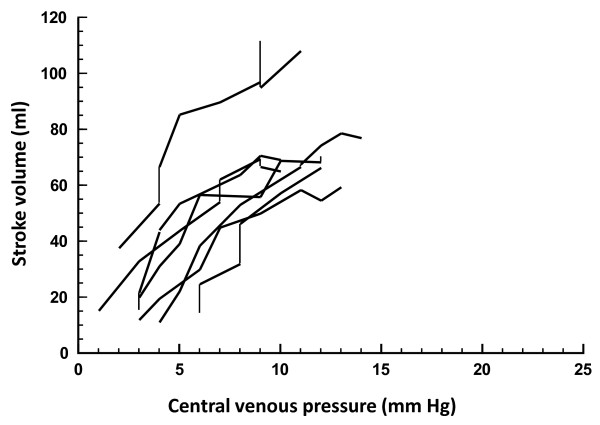
**Cardiac function curves showing fluid challenges in controls**. Changes in stroke volume are shown in relation to concomitant changes in central venous pressure. Connected lines represent subsequent fluid challenges in individual animals.

**Figure 2:**
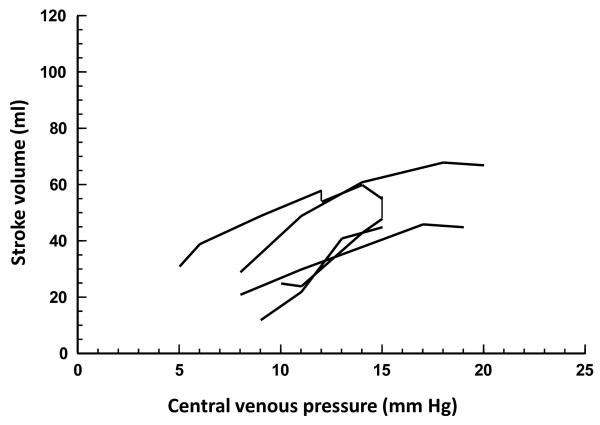
**Cardiac function curves showing fluid challenges in endotoxemia preceding hemorrhage**. Changes in stroke volume are shown in relation to concomitant changes in central venous pressure. Connected lines represent subsequent fluid challenges in individual animals.

**Figure 3:**
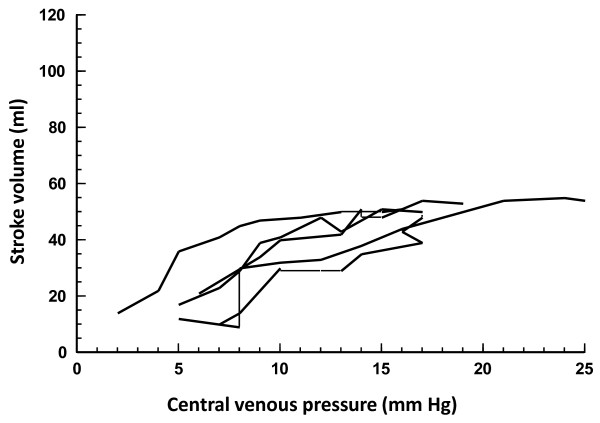
**Cardiac function curves showing fluid challenges in endotoxemia after bleeding during retransfusion and volume expansion**. Changes in stroke volume are shown in relation to concomitant changes in central venous pressure. Connected lines represent subsequent fluid challenges in individual animals.

Fifty-one fluid challenges were performed in controls (Table 2), and 34 of these (67%) increased the stroke volume. If only fluid challenges that increased the CVP by > 1 mmHg are considered (n = 39), then 28 (72%) challenges increased the stroke volume. Table 3 summarizes the hemodynamics before and after the fluid challenges in responders and nonresponders in both groups of animals. The cardiac function curves under different conditions are displayed in Figures 1, 2 and 3.

**Table 3:** Systemic hemodynamic values and blood pressure variation before and after volume challenges

		Volume challenges during endotoxemia		Volume challenges in controls	
			
		Before	After	Before	After
HR	All	114 ± 15	113 ± 13	113 ± 16	114 ± 16
(beats/min)	Responders	115 ± 16	110 ± 14	111 ± 17	110 ± 16
	Nonresponders	113 ± 12	115 ± 13	116 ± 15	122 ± 13

MAP	All	90 ± 30	98 ± 26*	90 ± 30	100 ± 23*
(mmHg)	Responders	86 ± 31	99 ± 25	81 ± 30^†^	95 ± 23
	Nonresponders	96 ± 28	96 ± 28	108 ± 20	110 ± 19

CO	All	4.3 ± 1.4	4.8 ± 1.3*	5.8 ± 2.7	6.8 ± 2.6*
(l/min)	Responders	3.7 ± 1.3^†^	4.6 ± 1.2	4.6 ± 2.4^†^	5.9 ± 2.6
	Nonresponders	4.9 ± 1.3	5.0 ± 1.3	8.2 ± 1.5	8.5 ± 1.3

SV	All	38 ± 14	43 ± 12*	52 ± 24	59 ± 21*
(ml)	Responders	33 ± 13^†^	42 ± 12	42 ± 22^†^	54 ± 22
	Nonresponders	44 ± 12	44 ± 12	71 ± 14	71 ± 13

CVP	All	12 ± 4	13 ± 4*	7 ± 3	8 ± 3*
(mmHg)	Responders	10 ± 4^‡^	12 ± 4	6 ± 3^†^	7 ± 3
	Nonresponders	13 ± 4	14 ± 4	10 ± 3	11 ± 2

PAOP	All	11 ± 4	12 ± 4*	9 ± 4	10 ± 4*
(mmHg)	Responders	11 ± 3	12 ± 4	7 ± 3^†^	9 ± 3
	Nonresponders	12 ± 4	13 ± 5	12 ± 3	13 ± 3

PPV	All	13 ± 7	12 ± 6	11 ± 8	9 ± 4*
(%)	Responders	14 ± 7	12 ± 5	14 ± 8^†^	9 ± 5
	Nonresponders	12 ± 7	11 ± 6	7 ± 3	8 ± 4

Systemic hemodynamic values and blood pressure variation before and after volume challenges during endotoxemia (before and after bleeding) and in controls (after bleeding). Data presented as mean ± standard deviation. HR, heart rate; MAP, mean arterial blood pressure; CO, cardiac output; SV, stroke volume; CVP, central venous pressure; PAOP, pulmonary artery occlusion pressure; PPV, pulse pressure variation. *P < 0.005, compared with before. ^†^*P *≤ 0.001, compared with nonresponders; ^‡^*P *< 0.05, compared with nonresponders.

### Pulse pressure variation and volume responsiveness

**Figure 4:**
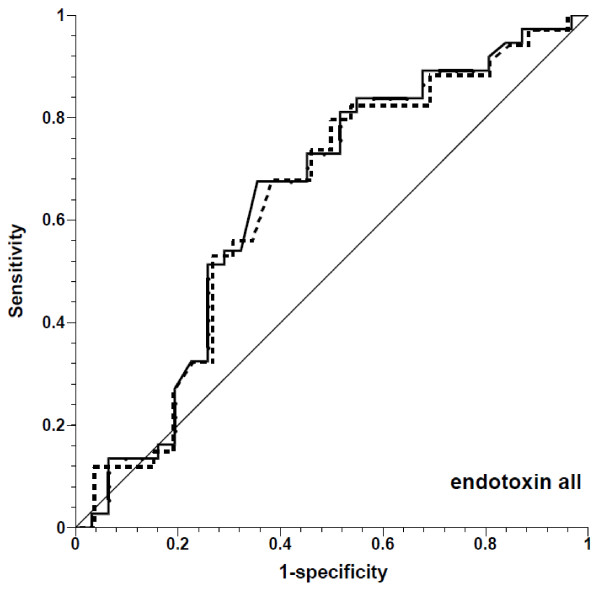
**Receiver operating characteristic curves for prediction of ≥10% increase in stroke volume by pulse pressure variation, showing all fluid challenges during endotoxemia**. Solid line, all fluid challenges; dashed line, fluid challenges with concomitant increase in central venous pressure; thin solid line, line of identity.

**Figure 5:**
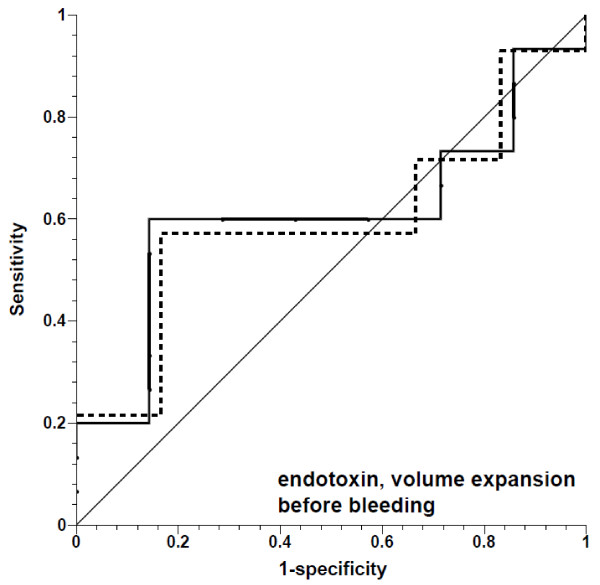
**Receiver operating characteristic curves for prediction of ≥10% increase in stroke volume by pulse pressure variation, showing fluid challenges in endotoxemia preceding hemorrhage**. Solid line, all fluid challenges; dashed line, fluid challenges with concomitant increase in central venous pressure; thin solid line, line of identity.

**Figure 6:**
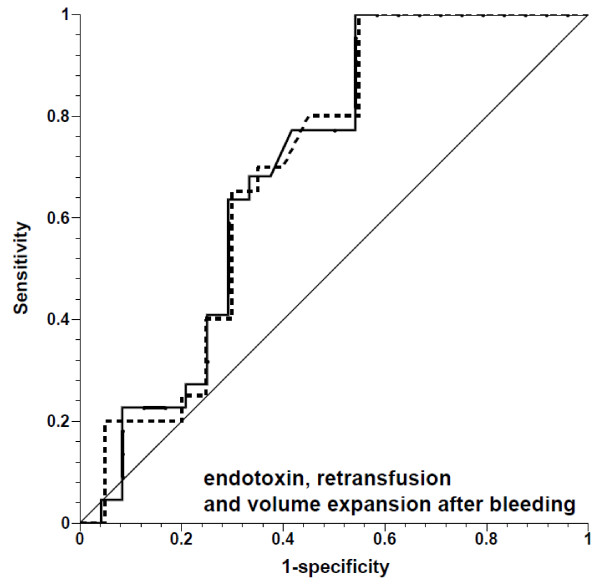
**Receiver operating characteristic curves for prediction of ≥10% increase in stroke volume by pulse pressure variation, showing fluid challenges in endotoxemia after bleeding during retransfusion and volume expansion**. Solid line, all fluid challenges; dashed line, fluid challenges with concomitant increase in central venous pressure; thin solid line, line of identity.

**Figure 7:**
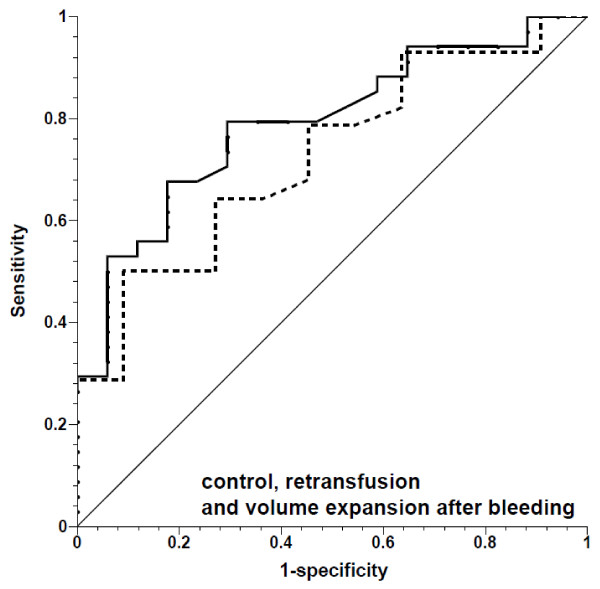
**Receiver operating characteristic curves for prediction of ≥10% increase in stroke volume by pulse pressure variation, showing fluid challenges in controls after bleeding during retransfusion and volume expansion**. Solid line, all fluid challenges; dashed line, fluid challenges with concomitant increase in central venous pressure; thin solid line, line of identity.

PPV was a poor predictor of an increase in stroke volume in endotoxemia, and did not predict volume responsiveness before bleeding. The area under the ROC curve (Figures 4, 5, 6 and 7) was 0.642 for all fluid challenges during endotoxemia (*P *= 0.045; Table 2), and this was related to the fluid challenges performed after bleeding (area under the ROC curve = 0.699, *P *= 0.021). In controls, PPV was a predictor of stroke volume increase (area under the ROC curve = 0.790, *P *= 0.001). Inclusion of only those fluid challenges with a CVP increase did not improve the prediction of increase in stroke volume.

The threshold values for best prediction (even if the area under the ROC curve was not significant) varied from 9 to 12% (Table 2). Using the cut-off value of 9% resulted in a sensitivity of 0.84 (95% confidence interval = 0.68 to 0.94) and a specificity of 0.39 (95% confidence interval = 0.22 to 0.58). The positive predictive value was 0.62 (95% confidence interval = 0.47 to 0.75) and the negative predictive value was 0.67 (95% confidence interval = 0.41 to 0.87).

Using a PPV threshold ≥13% resulted in a sensitivity of 0.46 (95% confidence interval = 0.30 to 0.63) and a specificity of 0.74 (95% confidence interval = 0.55 to 0.88). The positive predictive value was 0.68 (95% confidence interval = 0.46 to 0.85) and the negative predictive value was 0.53 (95% confidence interval = 0.38 to 0.69).

## Discussion

The main finding of the present study was that the predictive value of PPV for volume responsiveness is modified during an endotoxemia-induced acute increase in pulmonary artery pressure. In hemorrhage-induced hypovolemia, PPV could predict volume responsiveness, as expected. In contrast, during acutely increased pulmonary artery pressure in endotoxemia, the predictive value of PPV for volume responsiveness was lost. This is very similar to our finding of the poor predictive value of PPV for volume responsiveness in patients with elevated pulmonary artery pressure [13]. While our present findings provide proof for the concept that PPV may not predict volume responsiveness in the presence of pulmonary artery hypertension, there are relevant differences between the two studies and limitations that need to be considered.

First, in this experimental study, pulmonary artery hypertension was induced very acutely in pigs with previously healthy hearts; whereas in the clinical study, pulmonary hypertension was either due to sepsis or to pre-existing cardiac disease with mild to moderately elevated pulmonary artery pressure. In patients with sepsis, global myocardial dysfunction is likely to be present [15], and patients after cardiac surgery are likely to have postoperative myocardial dysfunction [16]; in addition, there is an increased risk of right ventricular dysfunction in the early postoperative period [17,18]. The consequences of acute changes in right ventricular loading were likely to represent those observed in patients, however, because pigs also demonstrated signs of acute heart dysfunction after endotoxemia.

Second, the magnitude of PPV between the studies was different: the mean PPV at baseline (13%) and before volume loading in the endotoxemic animals (10%) as well as in controls (11%) was comparable with values reported in healthy pigs [19,20] and in healthy dogs [21]. In the accompanying paper, the mean PPV in patients with septic shock (mean 27%) and in patients after cardiac surgery (mean 20%) was considerably higher, despite the use of a moderate tidal volume of 8 to 10 ml/kg [13]. These differences may be explained at least in part by species-related differences in the mechanical properties of the cardiovascular system, as well as in the thorax and the abdomen. The PPV values we found in patients were higher than in most studies previously reported in the literature. In cardiac surgery patients, PPV ranging from 11 to 15% (15 to 20% in responders) has been reported [22-30]; and in septic patients, PPV from 9 to 19% (13 to 24% in responders) [3,31-35]. As discussed in detail in the accompanying paper, the patients also had substantially higher pulmonary artery pressures than have been reported by others [13]. In the present study, the pulmonary artery pressure was increased even further.

Third, the cardiovascular effects of endotoxin are not limited to increased pulmonary artery pressure and acute right ventricular loading [36-38]. Endotoxin may impair the systolic and diastolic functions of both ventricles to a variable extent, and the overall impact may therefore also vary. Perhaps the most important difference between our two studies was that, despite the loss of the predictive value of PPV in both studies, the pigs remained volume responsive whereas most of the patients were nonresponders. This strongly suggests that different mechanisms may have been present to explain the poor predictive value of PPV. The lack of volume responsiveness in the patients was frequently associated with decreased right ventricular ejection fraction, suggesting impaired systolic function. In contrast, in the present study the pigs had severely reduced stroke volume and increased filling pressures but mostly preserved volume responsiveness. This suggests a relevant impairment of diastolic function and increased elastance. It is conceivable that systolic and diastolic dysfunction coexist to a variable extent, and their relevance to fluid responsiveness may also be modified by fluid challenges (for example, due to an acute septal shift).

Since we did not perform echocardiography, no conclusions on the exact mechanisms can be made. It should be acknowledged, however, that the contribution of Δup to the PPV (decrease in afterload with inspiration and squeezing of blood out of the lungs) is affected by the volume of blood in the lungs (likely to be higher in septic animals) and by the function of the left heart (likely to be more afterload-responsive in endotoxic animals). The afterload-reducing effect is related to how much pleural pressure rises with breaths, which will be increased if the chest wall compliance is reduced by edema (as expected with volume loading). This effect could be operative at higher volumes.

Another variable is tidal volume, which was kept constant. With decreasing chest wall and lung compliance, however, a constant tidal volume will be associated with increased pleural pressure swings. Besides affecting left ventricular afterload, the pleural pressure inhibits venous return, which should increase PPV. A complicating factor, however, is that pleural pressure also may increase the creation of zone II areas in the lung, which increases the afterload on the right ventricle and reduces the right ventricular stroke volume. This decrease is not volume responsive when the heart is functioning on the plateau of its function curve. This seems to be a likely explanation for our findings.

A further complicating variable is the abdominal reservoir, which increases when the animals are volume loaded as in our experiments. The descent of the diaphragm can therefore result in transfer of abdominal volume to the chest, and thus in an increase of right ventricular filling during inspiration.

These pathophysiological considerations demonstrate the complexities of PPV, which should be addressed in further studies. Nevertheless, our two studies clearly demonstrate that the predictive value of PPV for fluid responsiveness is lost under various conditions with increased pulmonary artery pressure. Although acute severe hypovolemia induced by bleeding restored some of the predictive value of PPV in endotoxemia, this is a rare clinical scenario. The high false-positive rate of PPV in predicting fluid responsiveness was recently shown by Mahjoub and colleagues [10]. Those authors considered it relevant enough to warrant echocardiography before fluid challenges are performed in patients with increased PPV.

Two limitations of the present paper, and a general limitation of the "PPV as a predictor of stroke volume response" approach, should briefly be addressed. First, the number of pigs in the present study is relatively small. In terms of fluid challenges, however, this study is certainly one of the largest. Second, PPV has been analyzed over only one respirator cycle in our study. Nevertheless, we did not find different values when analyzing PPV over five consecutive respiratory cycles at various time points in the experimental protocol.

The percentage of stroke volume increase has generally been used along with PPV as a criterion of volume response. With large variations of stroke volume, however, the requested percentage for a significant increase of stroke volume (usually 10%) may represent absolute changes that range from clinically highly significant to negligible.

Finally, we would like to acknowledge that the model we used for fluid administration was designed to test the ability of PPV to predict fluid responsiveness. In a clinical situation, double-checking the lack of response to a fluid challenge does not make sense.

## Conclusions

Our two studies suggest that both false-positive and false-negative values are common for PPV when the pulmonary artery pressure is increased. Increased pulmonary artery pressure is common in intensive care patients, especially in sepsis and after cardiac surgery, but may be overlooked unless echocardiography or the pulmonary artery catheter is used. We therefore strongly suggest caution in using PPV to predict volume responsiveness.

## Key messages

• PPV does not predict fluid responsiveness during endotoxin-induced pulmonary artery hypertension.

• During severe hemorrhage in endotoxemia, the predictive value of PPV is low.

• Volume challenges triggered by high PPV may lead to fluid accumulation in these situations.

## Abbreviations

CVP: central venous pressure; PPV: pulse pressure variation; ROC: receiver operating characteristic.

## Competing interests

The Department of Intensive Care Medicine holds, or has in the past held, research contracts with Abbott Nutrition International, B. Braun Medical AG, CSEM SA, Edwards Lifesciences Services GmbH, Kenta Biotech Ltd, Maquet Critical Care AB, Omnicare Clinical Research AG, and Orion Corporation, and holds or has held research and development/consulting contracts with Edwards Lifesciences SA and Maquet Critical Care AB. The money is/was paid into a departmental fund; no author receives/received individual fees. The past contract with Edwards Lifesciences is unrelated to and did not influence the current study.

## Authors' contributions

FD and DT carried out the animal experiments and analyzed a significant part of the data, and drafted the manuscript. SK participated in the experiments and analyzed the data. SMJ and JT designed and supervised the experiments, performed statistical analyses, and critically revised the manuscript.

## Acknowledgements

The authors would like to express gratitude to Olgica Beslac, Dr Daniel Mettler, and Daniel Zalokar for their skillful assistance during the experiments, and to Jeannie Wurz for editing the manuscript.
